# Surgical outcomes of intraabdominal versus vaginal approach for uterine manipulation during total laparoscopic hysterectomy

**DOI:** 10.1097/MD.0000000000033927

**Published:** 2023-06-02

**Authors:** Cenk Mustafa Güven, Dilek Uysal, Zafer Kolsuz, Bülent Yilmaz

**Affiliations:** a Department of Obstetrics and Gynecology, Izmir Private Can Hospital, İzmir, Turkey; b Department of Obstetrics and Gynecology, Atatürk Training and Research Hospital, University of Katip Çelebi, İzmir, Turkey; c Department of Obstetrics and Gynecology, Kütahya Health Sciences University, Kütahya, Turkey; d Department of Obstetrics and Gynecology, Recep Tayyip Erdoğan University, Rize, Turkey.

**Keywords:** gynecology, myoma screw, total laparoscopic hysterectomy, uterine manipulator, uterus

## Abstract

Uterine manipulation is essential for moving the uterus and proper anatomical dissection without complications during total laparoscopic hysterectomy (TLH). Although many different uterine manipulators (UM) have been designed in the last few decades, there is still no “optimal UM” that is universally safe, efficient, and cost-effective. This study aimed to compare myoma screw (MS) and UM with regard to surgical outcomes and cost-effectiveness in patients who underwent TLH. In the current study, we describe an operation technique that uses a MS instead of a uterine manipulator during TLH and discuss the surgical outcomes of this method. The use of MS resulted in significantly shorter operation time with respect to UM for uterine manipulation during TLH regarding benign indications, with affordable costs. The use of MS is a safe and cost-effective alternative to the use of UM during TLH.

## 1. Introduction

Every year, 1.500.000 women around the world undergo hysterectomy for the treatment of benign conditions, such as prolapse, abnormal bleeding and leiomyoma, or gynecologic cancers.^[1]^ Traditionally, hysterectomy was performed via laparotomy or the vaginal approach until the late 1980s when the first laparoscopic procedures were introduced.^[2]^ Since laparoscopic hysterectomy has some superiorities to other hysterectomy types, such as high patient satisfaction, earlier return to work, less blood loss, the possibility of diagnosing and treating other pelvic diseases, and the ability to maintain thorough intraperitoneal hemostasis, surgeons often preferred this approach and total laparoscopic hysterectomy (TLH) became the most frequently utilized method for hysterectomy during the last decade, particularly in developed countries.^[3,4]^ On the other hand, TLH also has a few shortcomings, including high cost, longer operation time, and the need for sophisticated technological tools such as sealing devices and uterine manipulators (UM).^[3–5]^

The primary aim of using a UM is to increase the distance between the cervix and ureter, thereby allowing safer dissection around the cervix and facilitating colpotomy by delineating the cervicovaginal junction.^[6]^ However, the literature lacks sufficient clinical evidence proving whether UM utilization fulfills these expectations.^[7]^ Besides, the use of UMs has been associated with several specific complications, including vaginal wall laceration, bowel perforation, and uterine rupture.^[8]^ There are also situations where UMs cannot be used, such as in patients with vaginal stenosis, anatomic variations that complicate identification of the uterine, cervix, and among those refusing vaginal penetration due to virginity.^[9]^

Although many different UMs have been designed in the last few decades, there is still no “optimal UM” that is universally safe, efficient, and cost-effective.^[7]^ Some researchers have proposed solutions to the problems encountered with UM use, including alternatives such as the use of specific sutures, grasping forceps, or myoma screws (MS).^[9–12]^ The MS is a standard reusable tool that is commonly used in myomectomy performed via laparotomy, laparoscopy, or the vaginal approach.^[13]^ MSs are known for their value in providing strong traction force in 3 dimensions.^[14]^ Furthermore, unlike UMs, the use of MS does not necessitate special expertise. In the literature, no data comparing the use of MS with UM in TLH. In this study, we aimed to compare MS and UM with regard to surgical outcomes and cost-effectiveness in patients who underwent TLH.

## 2. Materials and methods

### 2.1. Study design and study group

This is a single-center, retrospective study performed at Atatürk Training and Research Hospital, Katip Çelebi University, Izmir, Turkey. All procedures followed were in accordance with the local Ethics Committee of Atatürk Training and Research Hospital, Katip Çelebi University, Izmir, Turkey (20.06.2018-IRB #217). All protocols were conducted under the principles of the Declaration of Helsinki. Informed consent was obtained from all individual participants included in the study.

The initial cohort assessed for eligibility comprised 89 women who had undergone TLH (58 with UM and 31 with MS) for benign hysterectomy indications by senior surgeons at our center from 2013 to 2018. After the first analysis of patient data, 69 patients were finally enrolled, 45 in the UM group and 24 in the MS group. The indications for hysterectomy were benign conditions in all patients and included dysfunctional uterine bleeding, fibroid uterus, endometrial hyperplasia, adnexal mass, and high-grade cervical neoplasia.

### 2.2. Data collection

Preoperative assessments included the recording of demographic characteristics (age, body mass index, previous pelvic surgery, history, and gravida-parity), detailed bimanual pelvic examination, a transvaginal ultrasound, cervicovaginal smear, and endometrial sampling. Pelvic surgery history was considered positive in patients with previous cesarean section, myomectomy, adnexal surgery, or any surgical procedure in the pelvis.

The following data were also obtained and recorded from electronic medical records: intra- and postoperative complications, duration of the operation, length of hospital stay, uterine weight, and blood loss. Estimated blood loss was calculated based on the difference in hemoglobin level from baseline to the postoperative 24th hour. The duration of the operation was defined as the time between the first incision and the extraction of the umbilical trocar. The length of UM assembly was added to the operation time (7–8 minutes). The need for reoperation due to any reason, development of urinary system trauma, and bowel or major artery injuries were defined as major complications.

### 2.3. Surgical approach

The method used for uterine manipulation (UM or MS) was decided according to the surgeon’s preference or depending on whether a UM was available at the scheduled surgery date. In 6 patients, no UM was available; thus, an MS was utilized for uterine manipulation instead of performing an abdominal hysterectomy. In 10 patients, an MS was the first choice of the surgeon. In 8 patients for whom the UM approach had been chosen, the device could not be applied due to vaginal stenosis or excessive cervical distortion, necessitating the use of MS. In nulliparous patients, MS was the method of choice for surgeons as the uterine manipulation method. There were 2 types of UM in the hospital inventory during the study period, the RUMI® II/KOH-Efficient^TM^ (Cooper Surgical, Trumbull, CT) device and the Clermont-Ferrand® (CF) (Karl Storz Gmbh & Co., Tuttlingen, Germany) device. Since we did not focus on comparing different types of UMs, we did not create different UM groups. All patients fasted for at least 8 hours prior to the surgery; no other bowel preparation methods were employed.^[15]^ All TLH procedures were performed under general anesthesia while the patients were in the dorsal lithotomy position. A Foley catheter was placed for drainage and was kept in place until postoperative day 1. A single shot of the first-generation cephalosporin 1 g (2 g if the patient weighed 80 kg or more) was administered prophylactically.^[16]^

A UM was placed in the uterus at the beginning of the operation in the UM group. A gauze-filled surgical glove was placed into the vagina just before the colpotomy in the MS group. In both groups, pneumoperitoneum was achieved from the umbilicus with a Veress needle. After enlarging the umbilical incision, a 10-mm scope was placed in the abdomen, followed by giving a 20° Trendelenburg position. Following the routine abdominopelvic inspection, 5-mm trocar sheaths were placed in both lower quadrants, approximately 3 to 4 cm medial to the crista iliaca anterior superior on both sides. Another 5-mm sheath was placed on the midclavicular line bilaterally, approximately 4 cm above the lower sheaths. In the MS group, an MS was introduced into the abdominal cavity via the right-side lower 5-mm sheath. Before MS insertion, a 2 cm area of the uterine fundus was coagulated with bipolar cautery. The minor bleeding sites presenting during MS insertion were treated by bipolar cautery. Then, the uterus was positioned cephalad, and to the right with the UM or the MS. A tissue fusion device was inserted via the left-side lower sheath, and a grasping smooth forceps was inserted from each of the left and right upper sheaths in all patients. Following completion of these preparatory steps, the left round ligaments were sealed and transected, the anterior leaflet of the broad ligament was identified and cut until the bladder peritoneal fold, and the gray area on the posterior leaflet of the broad ligament was identified and opened appropriately. The broad ligament window was enlarged to obtain sufficient distance from the ureter, followed by dissection of the posterior leaflet of the broad ligament until the cervical insertion of the uterosacral ligament. Then the infundibulopelvic ligament or ligamentum ovaria proprium was coagulated and transected. The uterus was then positioned cephalad but to the left with the UM or the MS. The same steps were carried out on the right side of the uterus. After completion, the vesicouterine peritoneal fold was dissected, and the bladder was pushed caudally to expose the cervicovaginal fascia. The uterine vessels were identified, coagulated, and transected bilaterally. Colpotomy was started from the left vaginal angle to the right, close to the cervix in all patients (UM and MS). Colpotomy was performed by using a Harmonic scalpel (Ethicon EndoSurgery Inc., Johnson & Johnson Medical SpA, Somerville, NJ) After the uterus was completely detached, it was removed via the vagina. Then the vagina was closed with 0 polyglactin 910 Vicryl (Ethicon, Cincinati) interrupted sutures with the intracorporal knotting technique. Before removing trocars, meticulous hemostasis was ensured, and the presence of ureter peristalsis was confirmed.

All patients were ambulated between the 8th and 24th hours after the operation. At postoperative day 1, the Foley catheter was removed. The timing of patient discharge was recorded as postoperative day 1, postoperative day 2, or later. Patients demonstrating spontaneous micturition without retention, the regular passage of flatus, and self-mobilization were discharged.^[17]^

### 2.4. Statistical considerations

Data were analyzed with the Statistical Package for the Social Sciences (SPSS) software (version 21.0; IBM, Armonk, NY). Categorical data were expressed as absolute and relative frequencies (n, %), while numerical data were expressed as mean ± standard deviation (minimum–maximum). The Shapiro–Wilk test was used to examine the normality of distribution in numerical data. The Mann–Whitney *U* test was used to compare the 2 independent groups in terms of numerical variables. *P* values of < 0.05 were considered to be statistically significant.

## 3. Results

Demographics and baseline characteristics were similar in both groups (Table 1). Indications and history of previous surgery of patients was shown in Table 2. Operation time was found to be significantly shorter in the MS group than in the UM group (*P* = .007). The comparison of differences in pre and postoperative hemoglobin levels and the length of hospitalization did not show significant differences between the groups (Table 3). With regard to major complications, 1 patient in the UM group had ureterovaginal fistula, which was recognized on postoperative day 15 and treated with ureteral stenting, 1 patient in the MS group suffered from a 2-cm cystotomy intraoperatively and was treated with a double-layer, continuous, nonlocking intracorporeal suture (2.0 polyglactin 910, Vicryl^®^). Both patients had uneventful follow-ups after treatment. There were no cases of intestinal injury, large vessel injury, or major bleeding necessitating blood transfusion in either of the groups.

**Table 1:** Demographic and baseline findings of patients.

	Uterine manipulator			Myoma screw			*P* value
	Mean ± SD	Min	Max	Mean ± SD	Min	Max	
Age (yr)	51.4 ± 8.2	41	71	52 ± 8.7	37	71	.423
Body mass index (kg/m^2^)	28,9 ± 4.43	42.70	21.80	27 ± 4.21	20.60	41.70	.220
Gravida (n)	2.8 ± 1.7	1	8	3.2 ± 1.8	0	8	.198
Parity (n)	2.5 ± 1.5	1	8	2.5 ± 1.0	0	5	.591
Preoperative hemoglobin levels (g/dL)	12.2 ± 1.7	8.1	14.4	12.3 ± 1.7	9.4	15.3	.930
Uterine weight (g)	203 ± 79	77	590	219 ± 68	83	620	.320

**Table 2:** Indications and history of previous surgery of patients.

	Uterine manipulator	Myoma screw	Total
Indication	In 45 patient	In 24 patients	In 69 patients
Leiomyoma	20 (45%)	10(41%)	30 (43.5%)
Adnexal mass	3 (6%)	2 (8%)	5 (7%)
HGSIL	3 (6%)	1 (5%)	4 (5.7%)
DUB	13 (29%)	7 (30%)	20 (29%)
EH	6 (14%)	4 (16%)	10 (14.4%)
History of previous surgery	13 in 45 patients (35%)	7 in 24 patients (34.5%)	20 in 69 (34.5%)
Cesarean	8 (61.5%)	4	12
Myomectomy	2	2	4
Hysterotomy	1	0	1
Nongynecologic	2	1	3

DUB = dysfunctional uterine bleeding, EH = endometrial hyperplasia, HGSIL = high-grade squamous intraepithelial lesion.

**Table 3:** Comparison of peri and postoperative outcomes of the 2 groups.

	UM			MS			
	Mean ± SD	Min	Max	Mean ± SD	Min	Max	*P* value
Operation time (min)	210 ± 58	125	349	156 ± 52	55	222	0.007*
Length of hospitalization s(d)	3.6 ± 1.1	2	8	3.3 ± 1.0	2	6	0.413
The difference of pre-post hemoglobin levels (g/dL)	1.8 ± 0.2	0.6	4.3	1.8 ± 0.3	0.6	3.9	0.865

MS = myoma screw, UM = uterine manipulators.

* Statistically significant.

## 4. Discussion

This study mainly found that operation time was significantly shorter for patients who underwent TLH with the MS approach compared to the UM approach, with affordable cost. However, the other surgical outcomes (length of hospitalization, pre and postoperative Hb levels, and complications) were similar.

Although a UM offers many advantages during TLH, the most important of which is the prevention of ureteral injuries, currently, that are no UMs that are accepted to be ideal; that is, none of the available devices have the features of being nonconductive, quickly assembled, easily inserted in the uterus, capable of maintaining pneumoperitoneum, while also being safe and cheap.^[18]^ To this aim, different types of UMs have been designed for use in TLH.^[7,18–21]^ Studies that compared UMs in TLH highlighted their various advantages and disadvantages. In the literature, although CF and RUMI (the manipulators used in our study) seemed to be the most versatile manipulators, they were also reported to be difficult to assemble.^[7,8]^ MS does not require an assembling process, and therefore, we believe this is one of the reasons behind shorter operation times between the UM and MS groups in this study; however, the difference in surgical duration was far greater than assembly time, indicating that procedural time difference was evident.

The use of UMs has also been associated with some significant complications.^[8]^ In this study, the tip of the UM breached the uterus in 3 cases (2 CF, 1 RUMI) but did not cause any complications, such as bleeding or adjacent organ damage. In more severe cases, uterine rupture can cause catastrophic consequences like bowel perforation.^[22]^

Parallel to the new manipulator design efforts, some experts proposed techniques that do not use a UM. Boztosun et al reported that the “uterine rein technique” (placing the uterus in a rein manipulated with a grasper) is both safe and effective with its relatively simple use allowing the operation to be performed with 2 primary surgeons.^[10]^ Puntembekar and colleagues concluded that the “uterine hitch technique” (hanging the uterus to the anterior abdominal wall with a simple suture) was a practical, cheap, and reliable method for uterine manipulation during oncological surgery and eliminated the need for additional assistance for vaginal manipulation.^[23]^ In the aforementioned trials by Boztosun and Putembekar, the average times required for hitching were reported to be 11.13 minutes and 5 minutes, respectively. Although MSs have similar manipulation characteristics to these techniques, an MS can save more time because fixation can be performed within seconds. Putemebekar mentioned some MS-related issues, such as the peritoneal spread of tumor cells or pus in malignant conditions and bleeding from the puncture site.^[23]^ All of the cases in our study had benign conditions and were free of tumor spillage risk. We did not encounter any excessive bleeding from the MS puncture site.

In another study, the authors described a method of TLH without UM use.^[24]^ They employed grasping forceps for uterine manipulation and concluded that this technique was a feasible and safe approach. They emphasized that their technique offers particular benefits in some circumstances; for instance, in patients with vaginal stenosis or patients with a distorted cervix, in the absence of UM, and in early-stage cervical or endometrial cancer surgeries. However, using grasper forceps for uterine manipulation increases the likelihood of slipping during manipulation, possibly causing tissue breakup and bleeding (especially common at the uterine cornua).^[23]^

Although we used a different technique for uterine manipulation (MS), our study supports the notion that options other than UM can be used for uterine manipulation in patients scheduled for TLH, thereby facilitating the use of laparoscopy. In our study, 8 patients who were scheduled for TLH with UM were operated on with MS, due to excessively narrow vagina (4 patients) and distorted cervix (4 patients). Also, when UM devices were unavailable in our hospital inventory, either an MS was used (6 patients) or the patient had undergone TLH via laparotomy.

The uterine weights measured in this study were similar in the UM and MS groups. In the literature, TLH was advised as the route of hysterectomy in cases with a large uterus instead of laparotomy, because uterine weight is not a contraindication for TLH while it is a contraindication for vaginal hysterectomy. Maccio emphasized the usefulness of UMs for laparoscopic hysterectomy of large uteruses ranging from 300 to 5320 g.^[25–27]^ However, in another paper, the same author also mentioned that performing TLH without a UM could be possible in situations where UM use was unfeasible due to anatomical reasons.^[9]^ In our study, the largest uteruses in the UM and MS groups weighed 590 g and 620 g, respectively. Therefore, in our study, while we confirmed the utility of UM for patients with a large uterus, we also showed that using MS for uterine manipulation was safe and efficacious.

The use of various devices, which are mostly disposable, is a major contributing factor to poor cost-effectivity in TLH. The UMs are responsible for 8% of the total expense of the TLH procedure.^[5]^ However, an MS is a reusable and rather durable tool. We used Storz 26175BL^®^ myoma fixation device as an MS. Because of its reusable property, only 2 MSs were used for all subjects who had undergone TLH with MS (each costing around 300 US Dollars). Since the cost of a UM for each subject was around 60 US Dollars, it is evident that the MS approach is far better in terms of costs while demonstrating comparable safety and reliability.

In the literature, there are very few studies that mention the surgical outcomes of MS use in TLH. Magdum et al studied the effectiveness and complication profile of MS in TLH. They concluded that MS was one of the most effective and cheapest uterine manipulation devices that could be used in TLH.^[28]^ In their large-volume study, they also reported that complications associated with MS were as follows: intestinal trauma (15 patients; 0.54%) and minor bleeding from the MS site (44 patients; 1.5%). Furthermore, Putembekar mentioned that their TLH technique (using an MS in all cases) is a well-tolerated and efficient surgery. Irrespective of previous surgery and uterine size.^[29]^ In our study group, we did not encounter any cases of intestinal damage or other major complications that could be directly attributed to MS use. MS puncture site bleeding events were minimal and controlled easily by bipolar cauterization.

## 5. Conclusions

In conclusion, the use of MS resulted in significantly shorter operation time with respect to UM for uterine manipulation during TLH regarding benign indications. Moreover, MS seems to be more ergonomic and cheaper in daily practice. Further randomized large-scale trials comparing the use of UM and MS in TLH are needed.

## Author contributions

**Conceptualization:** Cenk Mustafa Güven.

**Data curation:** Cenk Mustafa Güven, Dilek Uysal.

**Formal analysis:** Dilek Uysal.

**Investigation:** Cenk Mustafa Güven, Dilek Uysal.

**Methodology:** Cenk Mustafa Güven, Bülent Yilmaz.

**Resources:** Cenk Mustafa Güven, Dilek Uysal, Zafer Kolsuz.

**Software:** Zafer Kolsuz.

**Supervision:** Cenk Mustafa Güven, Bülent Yilmaz.

**Validation:** Cenk Mustafa Güven, Dilek Uysal, Zafer Kolsuz.

**Visualization:** Cenk Mustafa Güven, Dilek Uysal, Zafer Kolsuz.

**Writing – original draft:** Cenk Mustafa Güven.
